# Fluorescence angiography-assisted debridement of critically perfused glabrous skin in degloving foot injuries: Two case reports

**DOI:** 10.1097/MD.0000000000026235

**Published:** 2021-06-04

**Authors:** Mauro Vasella, Marco Guidi, Matthias Waldner, Maurizio Calcagni, Pietro Giovanoli, Florian S. Frueh

**Affiliations:** Department of Plastic Surgery and Hand Surgery, University Hospital Zurich, University of Zurich, Zurich, Switzerland.

**Keywords:** angiography, fluorescence, foot, indocyanine green, injury, plastic surgery

## Abstract

**Rationale:**

Degloving foot injuries are challenging to treat and associated with life-long sequelae for patients. An appropriate debridement of ischemic soft tissues with maximal preservation of glabrous skin is key during the reconstruction of these injuries. Indocyanine green (ICG) fluorescence angiography is an established technique for the intraoperative evaluation of tissue perfusion.

**Patient concerns:**

Two patients sustained complex foot injuries in traffic accidents, including multiple fracture dislocations and extensive degloving of the plantar skin.

**Diagnosis:**

Clinical inspection revealed significant degloving of the glabrous skin in both patients.

**Interventions:**

After fracture fixation, ICG fluorescence angiography-assisted debridement with immediate latissimus dorsi free flap reconstruction was performed.

**Outcomes:**

In both cases, this technique allowed a precise debridement with maximal preservation of the glabrous skin. The healing of the remaining glabrous skin was uneventful and the 6-month follow-up was characterized by stable soft tissues and satisfying ambulation.

**Lessons:**

ICG fluorescence angiography is a safe, user-friendly, and quick procedure with minimal risks, expanding the armamentarium of the reconstructive surgeon. It is highly useful for the debridement of extensive plantar degloving injuries and may also help to minimize the number of procedures and the risk of infection.

## 1 Introduction

Degloving foot injuries represent a rare challenge for reconstructive surgeons and are generally associated with life-long sequelae for the patient.^[1,2]^ From an anatomical point of view, degloving is defined as an avulsion of the cutaneous and subcutaneous layers resulting in the exposition of deeper tissues, such as fascia, muscle, or bone. Soft-tissue degloving is the consequence of run-over accidents with significant shearing forces, commonly encountered in traffic or farm accidents.^[1–4]^

A crucial step during the reconstruction of degloving foot injuries is an appropriate debridement of devascularized soft tissues. In clinical practice, evaluation of tissue vascularization is mainly based on the visual assessment of bleeding and vessel morphology, that is, vascular occlusion. However, clear demarcation is often delayed, and intraoperative assessment can be demanding and depends on a surgeon's experience, especially when assessing the perfusion of glabrous skin in complex foot injuries. The correct debridement of the plantar foot is particularly challenging because (i) the clinical assessment of perfusion may be difficult due to the thickness of the skin in this area and (ii) the unique type of skin cannot be adequately replaced and, therefore, it would be desirable to resect as little as possible.

The importance of glabrous skin preservation is highlighted by its biological characteristics. The epidermal layer is significantly thicker with ∼1.4 mm when compared to the integument of other anatomical areas.^[5,6]^ Moreover, numerous fibrous septae anchor the cutaneous tissue to the plantar aponeurosis, resulting in lower shearing forces during ambulation and lobules of subcutaneous adipose tissue serve as an optimal shock absorbent.^[7,8]^ Finally, the plantar sensitivity allows precise pressure detection, which is essential for weight redistribution while standing.^[5]^

Indocyanine green (ICG) fluorescence angiography is an established tool for the intraoperative assessment of tissue perfusion.^[7,8]^ It is a dye emitting diffuse fluorescence when exposed to near-infrared light with a wavelength ranging from 600 to 900 nm. Its half-life is ∼4 minutes with rapid binding to plasma proteins^[9]^ and it is eventually secreted into the bile. In this article, we used ICG fluorescence angiography for a more objective resection of critically perfused glabrous skin in two cases of foot reconstruction.

## 2 Case reports

### 2.1 Ethics approval and consent for publication

According to the local ethics committee, this study did not require specific ethical approval (BASEC-Nr. Req-2020-01293). Both patients gave written informed consent for the publication of their clinical images.

### 2.2 Technique of fluorescence angiography using ICG

The protocol involves the intravenous administration of 1 mL ICG dye with a concentration of 5 mg/mL (VERDYE; Diagnostic Green, Aschheim-Dornach, Germany) followed by a 10 mL saline bolus. It typically takes 15 to 30 seconds to see the full effect of fluorescence.^[10,11]^ Visualization and video recording is then performed using a near-infrared sensitive camera system (FLUOBEAM 800; Fluoptics, Grenoble, France). It is advisable to dim the light in the operating room before employing the camera system so that an optimal interpretation of the visualization on-screen is ensured. Well-perfused areas are displayed as bright white and hypo- or non-perfused areas appear dark on the screen. The process should not delay surgery for more than ∼5 minutes.

### 2.3 Case 1

**Figure 1:**
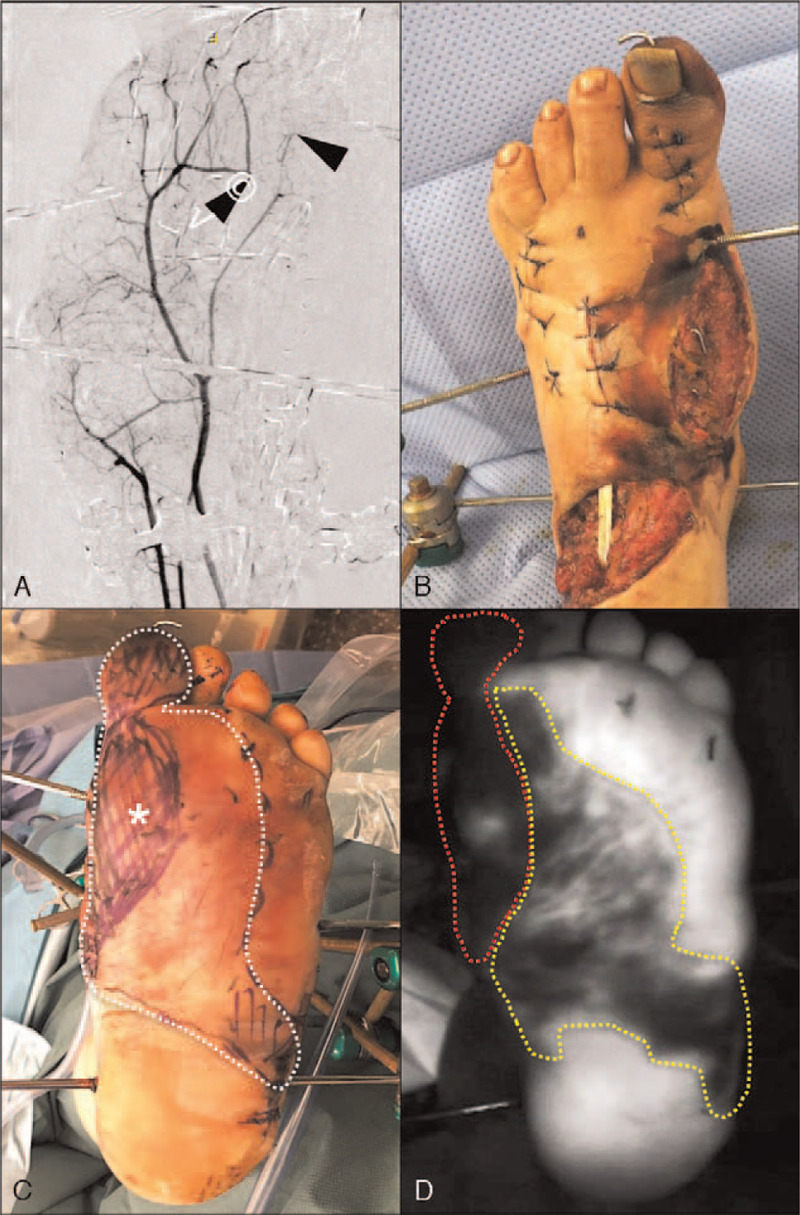
Great toe devascularization and partial plantar ischemia. (A) Preoperative angiography indicating a devascularized great toe with traumatic occlusion of the digital arteries (arrowheads). (B) and (C) Intraoperative clinical assessment of dorsal (B) and plantar (C) skin ischemia. Compared with the dorsal skin, the vascularization of the degloved glabrous skin (C, dashed line) is challenging to evaluate. Asterisk = resected area. (D) intraoperative ICG angiography revealing areas of complete (dashed red line) and partial (dashed yellow line) devascularization. ICG = indocyanine green.

**Figure 2:**
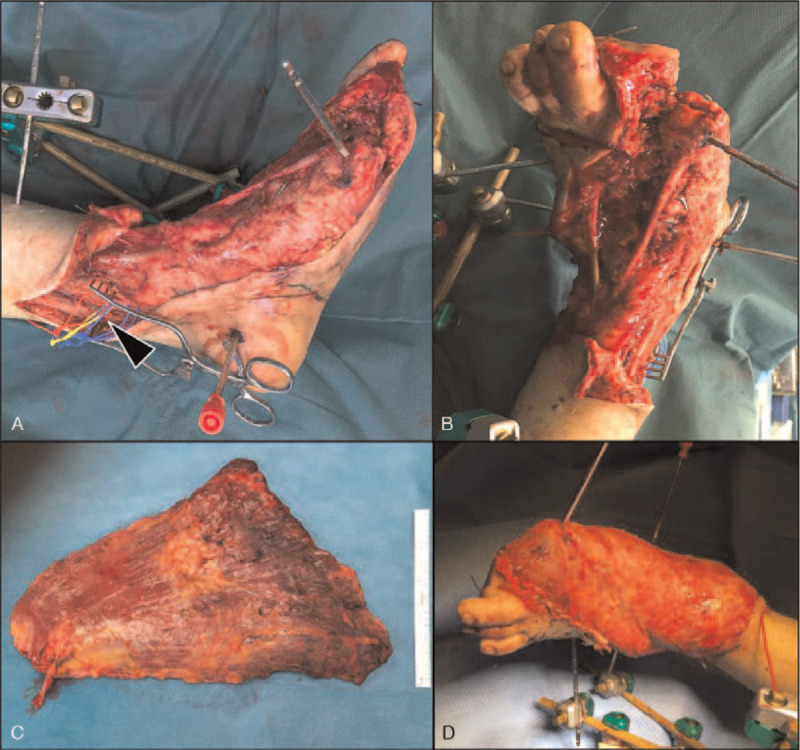
Debridement and soft tissue reconstruction. (A) Medial and (B) dorsal intraoperative pictures after debridement of devascularized tissues and dissection of the posterior tibial neurovascular bundle (A, arrowhead). (C) and (D) Latissimus dorsi flap before and after microvascular anastomoses and inset.

**Figure 3:**
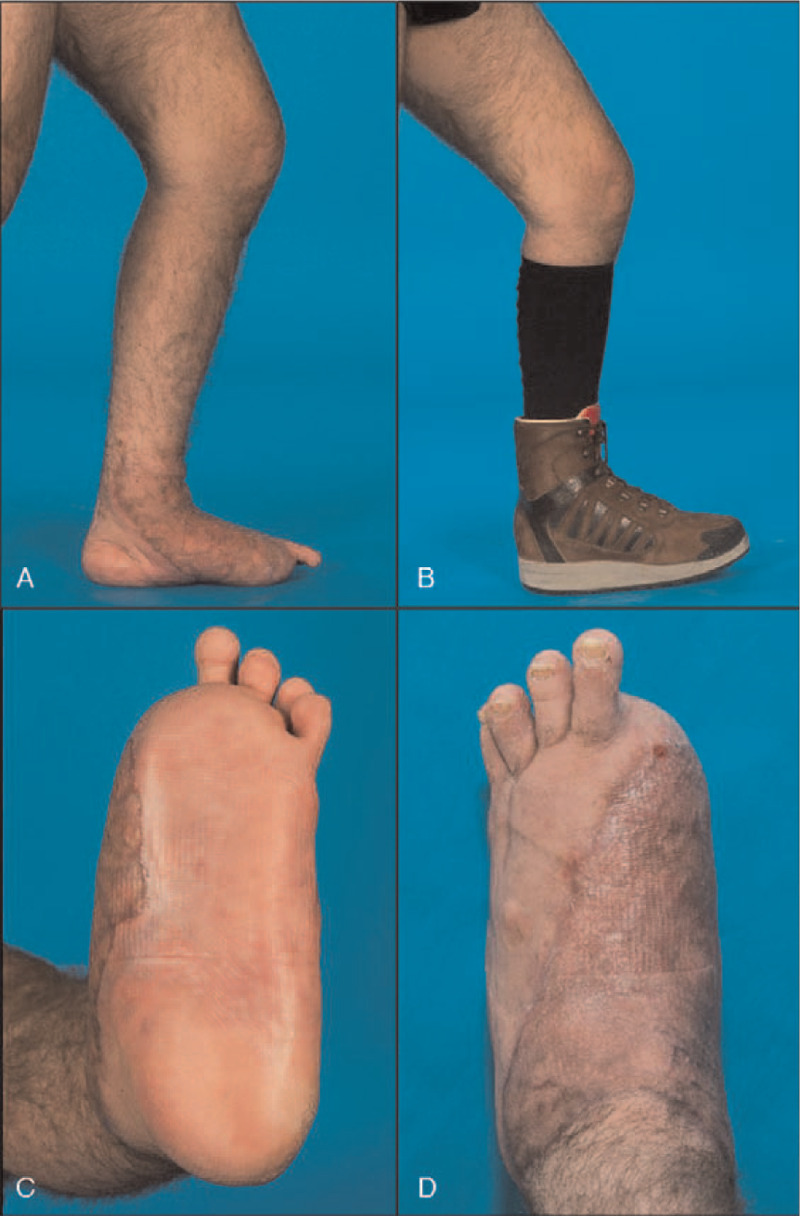
Clinical outcome after foot reconstruction. (A) and (B) Medial view of the reconstructed foot without and with orthopedic footwear illustrating good function in the upright position. (C) and (D) 6-month follow-up with a detailed view of the plantar skin showing only minimal scarring of the critically perfused glabrous skin.

The first patient, a 33-year-old male motorcyclist, was involved in a traffic accident with a truck. He sustained a polytrauma including a severe injury to his left foot. The lower extremity trauma consisted of tarsal and metatarsal fracture-dislocations and an extensive soft tissue injury with plantar degloving. Conventional angiography revealed occlusion of the arteries to the great toe with subsequent ischemia (Figs. 1A and B). Nine days after the accident, soft tissue reconstruction with a latissimus dorsi flap/skin graft was performed. However, the intraoperative assessment of the degloved glabrous skin was challenging due to unclear clinical demarcation (Fig. 1C). For a more objective debridement, intraoperative ICG fluorescence angiography was used (Fig. 1D). After great toe amputation and fluorescence angiography-assisted skin resection, the defect was covered with a latissimus dorsi flap anastomosed to the posterior tibial vessels under microscopic magnification (PENTERO 900; Carl Zeiss AG, Feldbach, Switzerland) (Fig. 2). Six months after surgery the sole of the foot remained covered by glabrous skin with good function, in particular normal sensation and stability to shearing forces. The patient reported good function of the reconstructed foot even being capable of hiking again in the Swiss alps (Fig. 3).

### 2.4 Case 2

**Figure 4:**
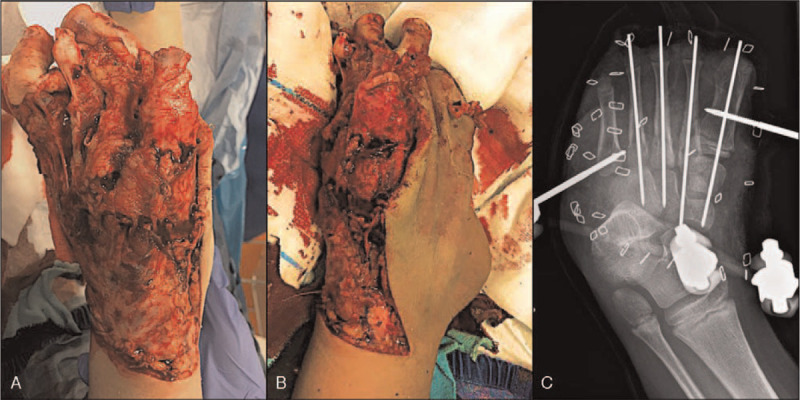
Pediatric degloving foot injury. (A) and (B) Preoperative dorsal and medial view revealing devascularized toes and extensive dorsal and plantar bone and soft tissue defects. (C) X-ray after toe amputation and temporary bone stabilization with K-wires and external fixation.

**Figure 5:**
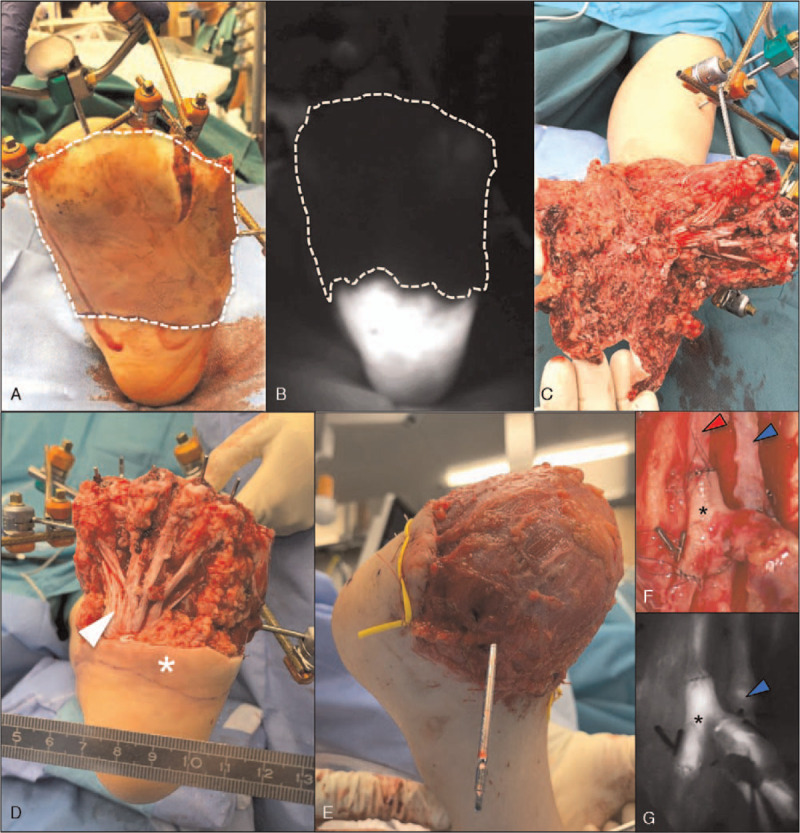
ICG angiography-assisted debridement and soft tissue coverage. (A) Intraoperative clinical assessment of ischemic glabrous skin (dashed line). (B) ICG fluorescence angiography identifying the completely devascularized area (dashed line) with higher precision. (C) and (D) Intraoperative photographs before and after debridement of ischemic soft tissues illustrating the plantar aponeurosis (arrowhead). Asterisk = critically perfused but preserved glabrous skin. (E) Latissimus dorsi flap after microvascular anastomosis and inset. (F) and (G) T-shaped flow-through anastomosis of the flap artery (asterisk) to the posterior tibial artery (red arrowhead). Blue arrowhead = comitant vein. ICG = indocyanine green.

**Figure 6:**
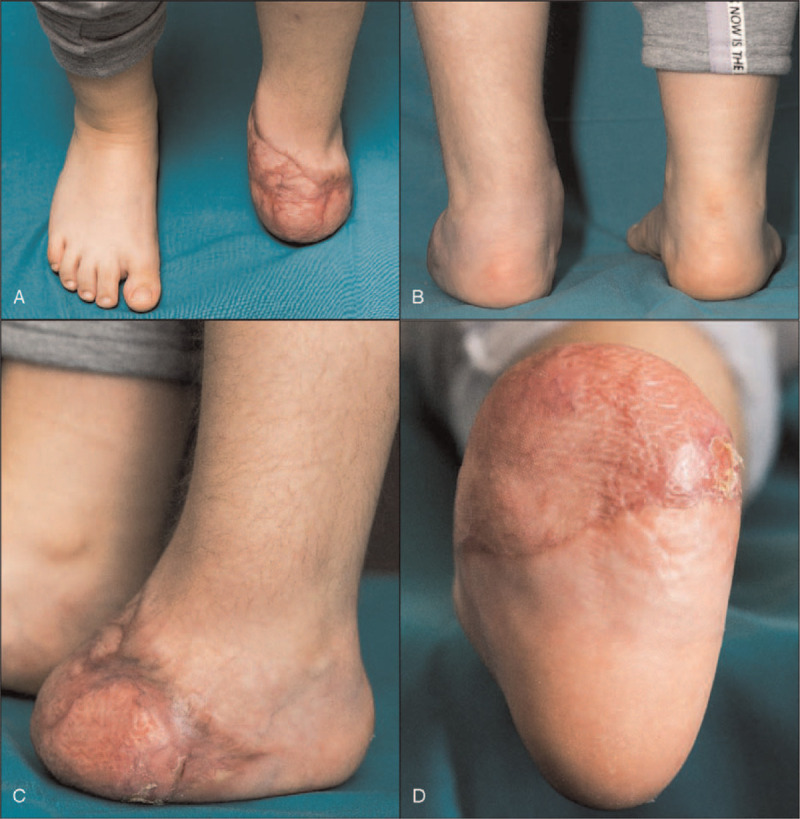
Clinical outcome after foot reconstruction. (A) to (C) Photographs 6 months after surgery. (D) Uneventful healing of the preserved glabrous skin and the grafted latissimus dorsi flap.

The second patient, a 6-year-old girl, sustained a severe foot injury in a run-over accident by a bus, resulting in subtotal toe amputation with multiple metatarsal fractures and an extensive dorso-plantar soft-tissue defect characterized by degloving of the planta pedis (Figs. 4A and B). Primary care consisted of toe amputation and K-wire osteosynthesis of the metatarsal fractures as well as external fixation and negative-pressure wound therapy (Fig. 4C). During the subsequent reconstructive surgery, the clinical assessment revealed impaired vascularization of the degloved glabrous skin (Figs. 5A and C). ICG fluorescence angiography-assisted debridement was performed to preserve only well-vascularized parts of the degloved plantar skin (Figs. 5B–D). Beside skin ischemia, the ICG angiography also revealed a devascularized fourth metatarsal following fracture of its base. After transmetatarsal amputation, the stump was covered with a microvascular latissimus dorsi flap anastomosed to the posterior tibial vessels and a meshed skin graft (Figs. 5E–G). At 6 months the healing of the flap was uneventful and the remaining glabrous skin stable (Fig. 6).

## 3 Discussion

ICG is a water-soluble tricarbocyanine dye that has been used for decades to measure cardiac output^[12]^ and liver blood flow.^[13]^ In clinical routine, ICG-based fluorescence angiography or lymphangiography is applied in different fields of reconstructive surgery. For instance, the use of ICG is popular to evaluate tissue perfusion during breast reconstruction,^[14,15]^ burns,^[16]^ and more recently, microsurgical procedures for chronic lymphedema or lymphoceles.^[17–19]^ However, only a few authors have reported the use of ICG fluorescence angiography during the reconstruction of lower extremity trauma.^[20,21]^ This is somewhat surprising because ICG fluorescence angiography-assisted debridement of critically perfused skin offers several advantages, especially in the case of plantar degloving.

Extensive degloving of the glabrous plantar skin is a rare and difficult injury and requires a sophisticated reconstructive plan. Importantly, the surgeon has to find a balance between aggressive debridement of devascularized skin and preservation of the biomechanically unique glabrous skin. The clinical assessment of skin perfusion can be particularly challenging in cases of subtotal degloving of the plantar foot as presented in Figure 5. In such a case, a significant amount of the degloved glabrous skin may still be axially vascularized by the plantar vessels, allowing partial preservation. Remarkably, skin resection based on clinical assessment alone would have resulted in over-debridement in the herein presented cases as indicated in Figures 1 and 5. Hence, it may be assumed that, despite occlusion of the major arterial vasculature, the rich collateral perfusion of the glabrous skin exerts protective effects even in severe degloving. Furthermore, this technique allows for an early assessment of skin perfusion, making a definitive and one-staged debridement possible. This should reduce the delay of definitive soft tissue coverage, the risk of infection, and in cases of open fractures, the risk of non-union.^[22]^ From the patient's perspective, fewer operative procedures are clearly beneficial and from an economical perspective, healthcare institutions may profit from a shorter total number and time of surgeries and length of hospital stay.

ICG fluorescence angiography is a safe, user-friendly, and quick procedure with minimal risks of anaphylactic reactions.^[23]^ Beside skin perfusion, it is also suitable to assess the vascularization of deeper structures, such as adipose tissue, muscle, or even bone.^[21,24]^ Moreover, it is useful for intraoperative evaluation of microsurgical anastomoses and free flap perfusion. In theory, it allows quantification of tissue vascularization through evaluating the percentage of maximal perfusion (ie, the perfusion of the adjacent normal skin). In our experience, however, we have used it as a semi-quantitative adjunct to clinical assessment in cases of unclear demarcation with convincing clinical results. Taken together, ICG fluorescence angiography is a nice tool expanding the armamentarium of the reconstructive surgeon and is highly useful for the decision-making during an accurate debridement and soft tissue coverage of extensive plantar degloving before obvious demarcation, reducing the number of interventions and the risk of infection.

## Author contributions

**Conceptualization:** Florian S. Frueh.

**Data curation:** Mauro Vasella, Marco Guidi, Florian S. Frueh.

**Surgery:** Florian S. Frueh, Matthias Waldner, Maurizio Calcagni, Pietro Giovanoli.

**Visualization:** Mauro Vasella, Florian S. Frueh.

**Writing – original draft:** Mauro Vasella, Florian S. Frueh.

**Writing – review & editing:** Marco Guidi, Matthias Waldner, Maurizio Calcagni, Pietro Giovanoli.
